# Merging microencapsulated garlic extract as a bioactive ingredient into manufacturing functional soft cheese

**DOI:** 10.1038/s41598-025-97107-y

**Published:** 2025-04-26

**Authors:** Tarek Nour Soliman, Ahmed Behdal Shazly, Mahmoud Abd El-Aziz, Laila Khaled Hassan

**Affiliations:** Dairy Department, Institute of Food Industries and Nutrition Research, National Research Centre, P.O. 12622, Giza, Egypt

**Keywords:** Encapsulated garlic extract, Whey protein isolate/Gum Arabic complex coacervate, Soft cheese, Nutrition, Quality of life, Biochemistry

## Abstract

The study aimed to produce a functional soft cheese with garlic extract (GE). First, the GE was encapsulated using gum Arabic (GA) and whey protein isolate, and its characteristics [zeta-potential, particle size, Coacervates yield, Encapsulation efficiency and polydispersity index, scanning electron microscope (SEM)] were evaluated. The free GE (FGE) and encapsulated GE (EGE) were then added to UF-soft cheese formulation (0.2 and 0.4% w/w). The samples were kept for 30 days in a refrigerator and their physicochemical, sensory properties, Texture profile, SEM and radical-scavenging activity were examined. EGE UF-soft cheese exhibited more free-radical scavenging activity than FGE at the same concentration and control, 25.14, 22.12, and 13.64%, respectively. The EGE-fortified cheese had a harder and gummier texture, a more noticeable change than the FGE and control cheese. In comparison to FGE-fortified and control, the sensory attribute representing overall quality, especially that fortified with 0.2% EGE displays the highest value. The study demonstrates that the use of EGE in cheese fortification provides a healthy and promising approach to reducing the strong flavor of garlic and enhancing bioactivity in the production of functional soft cheese, offering up novel opportunities for the development of distinctive dairy products with functional features.

## Introduction

Plant-derived essianals, such as herbs and spices, were utilized for taste and flavoring substances, preservatives, and therapeutic ingredients as resulting from their antibacterial and antioxidant components^1^. Garlic (*Allium sativum* L.), was consumed since the earliest times as a spice and a medicinal ingredient in many cultures due to its diverse of health biological activity^2^. It was represented in bioactive components such as flavonoids, phenols, polysaccharides, saponins, and substances containing organosulfur^3^. Garlic’s major active components are organosulfur compounds such as allicin (~ 75% of thiosulfinate component), alliin, allyl sulphide, (E)-ajoene, (Z)-ajoene, and 1,2-vinyldithiin^4^. The odour and flavor of garlic are caused by organosulfur compounds^5^. Garlic’s phenolic and organosulfur components are responsible for many of its biological and therapeutic properties, including its anticancer, anticoagulant, antibacterial, hypoglycemic, anti-inflammatory, cardioprotective, and immunomodulatory functions^4^. However, when exposed to environmental variables such as high temperatures, oxygen and light, organosulfur compounds decompose or oxidize, leading to the loss of their biological activity^3,6^. The microencapsulation technology is the most common and widely used in food industries as protection and preservation of bioactive ingredient for enhancing the availability; however, currently, the interest in microencapsulation technology increased due to their higher potential to enhance bioavailability, better controlled release of the encapsulated core material and higher precision targeting of the bioactive compounds as compared with Plant-derived extract bioactive ingredient as free^3,7^. Several encapsulation strategies, including coacervation, molecular inclusion, and cocrystallization, can be employed to protect the bioactive compounds from harmful interactions with other substances, increase stability to temperature, moisture, oxidation, and light, control the release of the encapsulated constituents, and covers undesirable flavors and odors^8^. Timilsena et al.^9^ mention to complex coacervation is a highly successful microencapsulation process used in a variety of industries, including the textile, agricultural, pharmaceutical, and culinary industries. During the procedure of coacervation, spontaneous liquid–liquid phase separation takes place as a result of biopolymers loaded with opposite charges interacting electrostatically^10^. Many variables have a significant impact, including ionic strength and charge density, concentration and mixing ratio of polymer with pH, and temperature, on the complicated coacervate process^11^. In the process of encapsulation, protein-polysaccharide complexes have been effectively formed using it^12^. Materials like polysaccharide and protein, which were the most often used wall materials in microencapsulation procedures, are required for the encapsulation process. Gum arabic is derived from the plant parts of acacia plants, a polysaccharide compound that has strong surface activity, high water solubility, high viscosity, and emulsification potential^13–15^. Whey protein isolate (WPI) is a whey protein product that is generated in amounts by the casein and cheese industries, is the second protein source, and has high functional characteristics in encapsulation. WPI is widely used in the food industry because of its excellent emulsifying properties by form fibrillar structures, under specific conditions, such as heat at low pH and ionic strength, and high nutritional value, and cost effectiveness^16^. Ultrafiltration is an approach to produce cheese which utilizes a membrane-based filtration system to concentrate and separate liquid ingredients according to their molecular size^17^. One of the best sources of dairy products is ultrafiltration (UF) cheese UF Soft cheese is a good source of essential nutrition for the human body due to containing higher levels of vital nutrients, such as vitamins, free fatty acids, minerals, lipids, and protein content and bioactive peptides, as well as lower lactose content compared to traditional soft cheese. Furthermore, UF soft cheese may help support muscle growth, reduce the risk of chronic diseases, and be a suitable alternative for individuals who have difficulty digesting lactose as mentioned by El-Sayed and Shazly^18^. Recent research has shown that adding plant extracts to cheese improves its flavor and texture, increasing nutrient content, and providing health benefits. Combining the plant extracts with cheese has received significant attention in health advertising and disease prevention in humans by delivering health-promoting factors and increasing total nutrient intake, thus improving diet quality^18,19^.

Garlic or its any of its derivatives can be included in a range of dishes, such as poultry, meat, beef, fish, etc., but their unpleasant flavor and susceptibility restrict their application in the dairy industry to degradation, volatilization, and oxidation during processing and storage. There aren’t many publications on the dairy industry’s use of garlic extract that has been encapsulated^8,20^. There has also been no research on the microencapsulation of garlic extract using a mix of gum Arabic and whey protein isolate, and then the freeze-drying technique is applied. Soft fresh cheese can be prepared in a variety of ways, preserved at low temperatures with or without a salt solution, and enhanced with a wide range of flavors and additions^21^. This could potentially attract consumers who are looking for novel and unique food products. As a consequence, the goal of this research was to (i) evaluate the characteristics of complex coacervation encapsulated garlic extract (EGE) based on a mix of WPI and gum Arabic (GA) as partition materials followed by freeze-drying; and (ii) evaluate the effect of its use on the properties of soft cheese compared to the free garlic extract (FGE) during storage at 5 ± 2 °C for 30 days.

## Materials and methods

### Materials

Whey protein isolates (90% protein, 4% moisture, 1% fat, and 3% ash), containing mainly β-lactoglobulin and α-lactalbumin, was purchased from Davisco Foods International (Le Sueur, USA). Gum Arabic (0.5% protein, 2.5% ash, and 7.0% moisture) was obtained from Merck Co (Darmstadt, Germany). Milk retentate (11.31% protein, 77.94% moisture, 13% fat, and 4.03% ash) was prepared UF unit at Animal Production Research Institute, Giza, Egypt. Two kinds of starter cultures (*Lactococcus lactis* spp. *lactis* and *Lactococcus lactis* spp. *cremoris*) and powdered rennet enzyme (Protease Rhizomucor miehei) were purchased from Danisco France SAS, 38,470 VINAY, France. A local market in Giza Province, Egypt was where the mature garlic *Allium sativum*, was obtained. The 2,2-diphenyl-1-(2,4,6-trinitrophenyl)-hydrazinyl (DPPH) was obtained from Sigma-Aldrich (St. Louis, MO, USA). HPLC-grade ethanol and methanol were obtained from Fisher Scientific UK (Bishop Meadow Road, Loughborough, Leics, LE11 5RG, UK). All chemicals and reagents from different suppliers were of analytical grade.

### Methods

#### Producing of garlic extract

Remove the outer skin from the ripe garlic cloves, then chop and grind in a mortar until they become a paste. After being soaked in a dark glass bottle (80% ethanol) and shaken for 12 h with an orbital shaker, Heidolph rotary shaker (Germany) at room temperature (25 ± 2 °C), the garlic paste was centrifuged for 30 min at 5000 rpm to obtain the supernatant as garlic extract, and the residue was twice extracted by using the same volume of ethanol. A rotating vacuum evaporator (made by ROTAVAPOR R110 company at Bauchi, Switzerland) was used to mix and evaporate all three of the ethanoic garlic extracts and recover the solvents at 50 °C^22^.

#### Coacervate microcapsules preparation

To construct microcapsules, four grams of WPI were dissolved in 100 ml of deionized water by utilizing an ultrasonic bath sonicator (Neytech model 28 H, USA) for 10 min at 25 ± 1 °C. After that, for 20 min at 40 °C, the materials were mixed using a mechanical stirrer (IKA^®^, Germany). Garlic extract in the amounts of 480, 720, and 960 mg was added, and the mixture was homogenized for 30 min at 4000 rpm with continuous stirring, adding 100 ml (2% gum arabic) solution. To expedite coacervation, a 1% citric acid solution was used to lower the pH to 3.75, and the mixture was stirred mechanically at 600 rpm until coacervates formed^23^. After that, they put the container in an ice bath and stirred the reaction mixture to cool it down. Since decantation was facilitated by storing the coacervated components at 7 ± 1 °C overnight, that temperature was maintained. Powdered microcapsules were obtained by lyophilizing the coacervates and storing them at 4 ± 1 °C until analysis.

*Coacervate yield* The coacervate yield was used to evaluate how the garlic extract ratio affected WPI/GA coacervate. The following equation was used to calculate the coacervate yield.

$$Coacervate\, yield\, (\% ) = \frac{{Dry\, weight\, ofcoacervates}}{{Total\, weight\, of\, WPI\, and\, GA\, in\, the\, \, solution}} \times 100$$

*Encapsulation efficiency (EE)*: Encapsulation efficiency is defined as the percentage of phenolic encapsulated in relation to the phenolic content initially added. The extraction of phenolic compounds from each FGE and EGE sample was performed as described by Rutz et al.^24^ with some modification. Garlic extract was dissolved in 50% aqueous acetone to ensure maximum solubility of the extract. Aliquots of 10 mg of EGE samples were dissolved in 4 mL of solvent mixture (methanol: acetic acid: water (50:8:42 v/v/v)), sonicated for 5 min, centrifuged for 15 min at 5000 rpm. These supernatants were used for analyzing total phenolic content according to the Folin-Ciocalteu method at UV light absorption 765 nm. The results were expressed as milligram gallic acid equivalent per gram of dry sample for TPC The encapsulation efficiency was calculated according to the equation below:

$$EE \, (\% ) = \left( {\frac{{(TP_{0} - TP_{S} )}}{{TP_{0} }}} \right) \times 100$$

TP_0_: Total phenolic contents in the sample before encapsulation, TP_S_: Total phenolic contents in the supernatant after centrifugation.

### Characterization of microcapsules

#### The mean particle size

To determine the average size of microparticles, the Mastersizer 2000 laser diffraction particle size analyzer (Malvern Panalytical, Worcestershire, WR14 1X, United Kingdom) was used. Dilution of sample with distilled water (1:100) was carried outbefore the size measurement at 25 °C. Three separate analyses of the distribution and average particle size were conducted.

#### Surface charge (Zeta potential)

Zeta potential of aqueous emulsion of microcapsules was measured by means of a Malvern Zeta sizerNano ZS with software DTS Ver. 4.10, (Malvern Panalytical, Malvern, United Kingdom) at 25 °C. Before analysis, emulsions were diluted to a particle concentration of 0.01% using distilled water.

#### Morphology of surface microcapsulat

A scanning electron microscope (SEM) was used to examine the surface’s morphology. Use double-sided tape with a piece of aluminum foil and slowly sprinkle ME powder onto the adhesive. The stubs were then coated with gold using a sputter coater to a thickness of roughly 300 A. Following that, the stubs were covered in gold using a sputter coater until they were about 300 A thick. Each sample was examined using a scanning electron microscope (Model-6100, Tokyo, Japan) at 2000× magnification and 10 kV accelerated voltage.

### Cheese making

UF-retentat to produce Fresh soft cheese was used as a method. Abd El-Aziz et al.^21^, Five portions of the retentate milk were divided equally in size. One batch was acted as a control, devoid of FGE or EGE, while four treatments. In order to same ratio of produce 0.2 and 0.4% GE (120 mg GE/g WPI/GA coacervate), garlic extract was added at rates of 0.2% and 0.4% as a free garlic extract (FGE) or at rates of 1.67 and 3.35% as an encapsulated extract (EGE). Following a 3% NaCl mixture, all batches of UF retentate milk were pastereted at 75 °C, cooled to 42 °C, and then 1% of each batch was infect inoculated with a mixed starting culture (1:1). The rennet enzyme (5000 UI) was added 0.03% to achieve coagulation in 40 min. 500 mL of UF milk retentate was transferred into plastic containers, then maintained at 42 °C until a stable coagulum formed. For 30 days, all cheese samples were kept at 5 °C.

#### Chemical analysis

According to the method AOAC^25^ the cheese samples were examined for total solids, total nitrogen (TN), fat, and ash. The proportion of TN was multiplied by 6.38 to get the protein content. A digital pH meter (HANNA, Instrument, Portugal) with a glass electrode was used to test the pH level. Cheese proteolysis was determined according to Innocente^26^ as ratio of the water-soluble nitrogen to total nitrogen (WSN/TN ratio). While water-soluble nitrogen was prepared by grinding 4 g cheese in a mortar with water at 50 °C until a homogeneous sample was obtained, and the paste was then diluted to 100 mL and filtered.

#### Radical-scavenging activity of cheese

Using a stable DPPH radical (DPPH^•^) test according to Brand-Williams et al.^27^, radical-scavenging activity of cheese samples was assessed in cheese supernatant made using the technique of Hassan et al.^28^. In a nutshell, 10 g of cheese samples were mixed with 10 mL of methanolic solution (1:4), and the resulting cheese mixtures were incubated at 40 °C for an hour before being centrifuged at 4000*g* for 5 min. One hunred mL of cheese supernatants were combined with 3.9 mL of DPPH working solution (25 mg/L methanol). After incubation for 30 min at 25 °C in the dark, the absorbance was measured at 517 nm. The test combination and a control solution DPPH solution without cheese supernatant were made in the same way. The following formula was used to determine the DPPH radical-scavenging activity:

$$Radical{\text{-}}scavenging activity of cheese \, ( \%)= \left[ {\left( {{{\text{A}}_0}- {{\text{A}}_{1}}} \right)/{{\text{A}}_0}} \right] \times 100$$

A_0_ is the absorbance of the control (DPPH solution), and A_1_ is the absorbance of the sample.

### Texture profile

#### Cheese microstructure

Cheese samples were cut into cubes (3 ± 0.5 mm) and fixed for two hours at 48 °C in 3% glutaraldehyde in a 0.05 M phosphate buffer at pH 7. The fixed cubes were dehydrated by soaking them in 30, 50, 70, and 95% ethanol for 20 min each, followed by two rinses with 0.05 M phosphate buffer. Then, cheese cube samples were washed with 100% ethanol at 48 and 58 °C. Following this, the cheese cubes were promptly dried in the critical point dryer (Samdri PVT-3B, Tousimis, Rockville, MD) for 5 h, according to Vardhanabhuti et al.^29^. After the surfaces of the produced cheese samples were vacuum coated with gold, and inspected using a scanning electron microscope (SEMJoel Jsm 6360LA, Japan)^30^.

#### Colour parameters

The CIE lab colour scale (Hunter, Lab Scan XE-Reston, VA, USA) was installed on a spectrocolorimeter (Tristimulus Colour Machine) in the reflection mode to measure the soft cheese’s colour parameters (L*, a*, and b*). L* denotes the range of blackness from zero to one hundred, a* the range of redness from plus one to zero, and b* the range of yellow from plus one to blue (–). Each time the instrument was calibrated, a white tile from the Hunter Lab Color Standard (LX No. 16379) was used; the values were as follows: X = 72.26, Y = 81.94, and Z = 88.14 (L* = 92.46; a* = − 0.86; b* = − 0.16).

#### Sensory evaluation

A panel of researchers of sensory taste from the department of dairy science at the Institute of Food Industries and Nutrition Research, Egypt, assessed the sensory attributes, including appearance, texture, taste, and overall quality. According to Wadhwani & McMahon, the cheese samples were assessed using a point hedonic scale, ranging (1–9) as strongly liking (9), liking (5), up to extremely detesting (1)^31^. Sliced into 1.5 × 1.5 × 1.5 cm cubes, the cheese samples were coated with plastic to keep them from drying out. The cubes were encoded with random three-digit numbers. Three cheese cubes were provided for each judge’s sample. They were given water and unsalted crackers in between samples to help clear their palates.

### Statistical analysis

Statistical analysis using SAS^32^ software was conducted using the GLM approach. ANOVA and Duncan’s multiple comparison method were used to compare the means. This study used triplicate methods for all of its experiments, and averages were taken. At *p* < 0.05, differences were considered significant.

## Results and discussion

### Microencapsulated garlic extract

#### Coacervate yield and efficiency

Table 1 shows the coacervate yield (%) and encapsulation efficiency (EE, %) of encapsulated garlic extract (EGE) using coacervate of gum Arabic (GA) with whey protein isolate (WPI). When FGE was added at a rate of 80 or 120 mg/g WPI/GA coacervate, the coacervate yield was comparable, but when 160 mg FGE was added, it was slightly reduced (*P* > 0.05). Action higher number of free amino sitesinteracts in the polymer backbone between positively charged (–NH^3+^) for chitosan and negatively charged carboxyl groups (–COO) of WPI as a result of an increase in the degree of deacetylation, which leads to higher coacervate yields^33^. Muhoza et al.^34^ observed similar findings in an earlier investigation. The WPI/GA coacervate showed a high EE of the EGE. However, the EE of EGE reduced from 96.78 ± 1.72% to 92.15 ± 2.37 and 84.75 ± 1.1% when the addition of FGE increased from 80 to 120 and 160 mg/g WPI/GA coacervate, respectively, indicating a dependence on the addition of FGE. Because garlic has better physical-chemical properties and more free amines to bond with WPI, more compounds may be preserved, which is primarily responsible for its ability to maintain phenolics^8^. However, Fraj et al.^35^ has previously reported that the encapsulation efficiency has dramatically decreased from 85.89 to 50.36% when essential oil concentration has increased.

**Table 1:** Coacervate yield, encapsulation efficiency, particle size, zeta-potential, and polydispersity index of encapsulated garlic extract using WPI/GA coacervate

Microcapsules characterization	Garlic extract concentration (mg/g of WPI/GA coacervate)		
	80	120	160
Coacervates yield (%)	71.65^a^ ± 2.25	71.50^a^ ± 2.35	68.39^a^ ± 1.80
Encapsulation efficiency (%)	96.78^a^ ± 1.72	92.15^b^ ± 2.37	84.75^c^ ± 1.09
Zeta-potential (mV)	− 22.00^a^ ± 1.50	− 19.65^b^ ± 0.55	− 18.60^b^ ± 0.75
Particles size (µm)	402^b^ ± 44	465^b^ ± 93	1156^a^ ± 623
Polydispersity index (PDI)	0.62^b^ ± 0.05	0.66^b^ ± 0.09	0.76^a^ ± 0.011

Means (*n* = 3 ± SD) with the same letters in the same row are not significantly different (*P* < 0.05)

*WPI/GA* whey protein isolate/gum Arabic

#### Microcapsules characterization

Table 1 represents the change in both particle size (nm), zeta potential (mV), and polydispersity index (PDI) of EGE, which were highly impacted (*P* < 0.05) by the FGE concentrations in the WPI/GA coacervate. As the concentration of GE increased, Zeta-potential decreased while PDI and particle size increased. At 160 mg FGE/g WPI/GA coacervate, nevertheless, the EGE’s particle size and PDI increased significantly. The mean particle size of WPI/GA coacervation was 402 ± 44, 465 ± 93, and 1156 ± 623 μm for varied ratios of 80, 120, and 160 mg GE, respectively. Sahoo et al.^36^ mention that microparticle size distribution is affected by several variables, including the core-to-wall balance, the preparation procedure, the stirring speed, the drying process, and the homogenization cycles. According to Rubio et al.^37^ PDI values of WPI/GA coacervate lie within the normal range (~ 0.7) of the size of particles as polydisperse. Similar results were reported by Fraj et al.^35^, who have reported the preparation of *Origanum vulgare* L.) essential oil nanocapsules showed PDI values of 0.19 and 0.31, respectively. In general, lower PDI values indicate high homogeneity in the particle population, the stability and narrow distribution of formulations^38^. However, the increase in PDI value can generate a relatively heterogeneous population of particles. Thus, the obtained results demonstrated that upon increasing the GE weight ratio, the particle size increases when other formulation variables are kept constant, thereby, suggesting that varying the core (essential oil) to wall (WPI/GA) materials weight ratio can affect the size and the PDI values. Zeta-potential increased from − 22.00 ± 1.50 mV at 80 mg GE to − 19.65 and − 18.60 mV at 120 and 160 mg/g of WPI/GA, respectively. According to Rocha-Selmi et al.^39^. In every complex coacervate, electrostatic interactions neutralized the biopolymer charges as expected. The zeta-potential values show that WPI and GA are likely to be combined and aggregate with opposing charges. The main reason for aggregations, which lead to creation of coacervates insoluble complex, is the absence of electrostatic repellence between biopolymers^40^. These findings aligned with previous research Ma et al.^41^, which indicated that as the concentration of ferulic acid increased, the absolute value of the zeta potential for the complex of mung bean globulin and ferulic acid increased. A dispersion system with a zeta-potential of − 30 mV or less suggests that the dominant interparticle interactions are repulsive^42^. Electrostatic interactions effectively neutralized the complexes’ net charges, as confirmed by a zeta-potential study on the dispersion WPI/GA.

#### Morphology

**Fig. 1:**
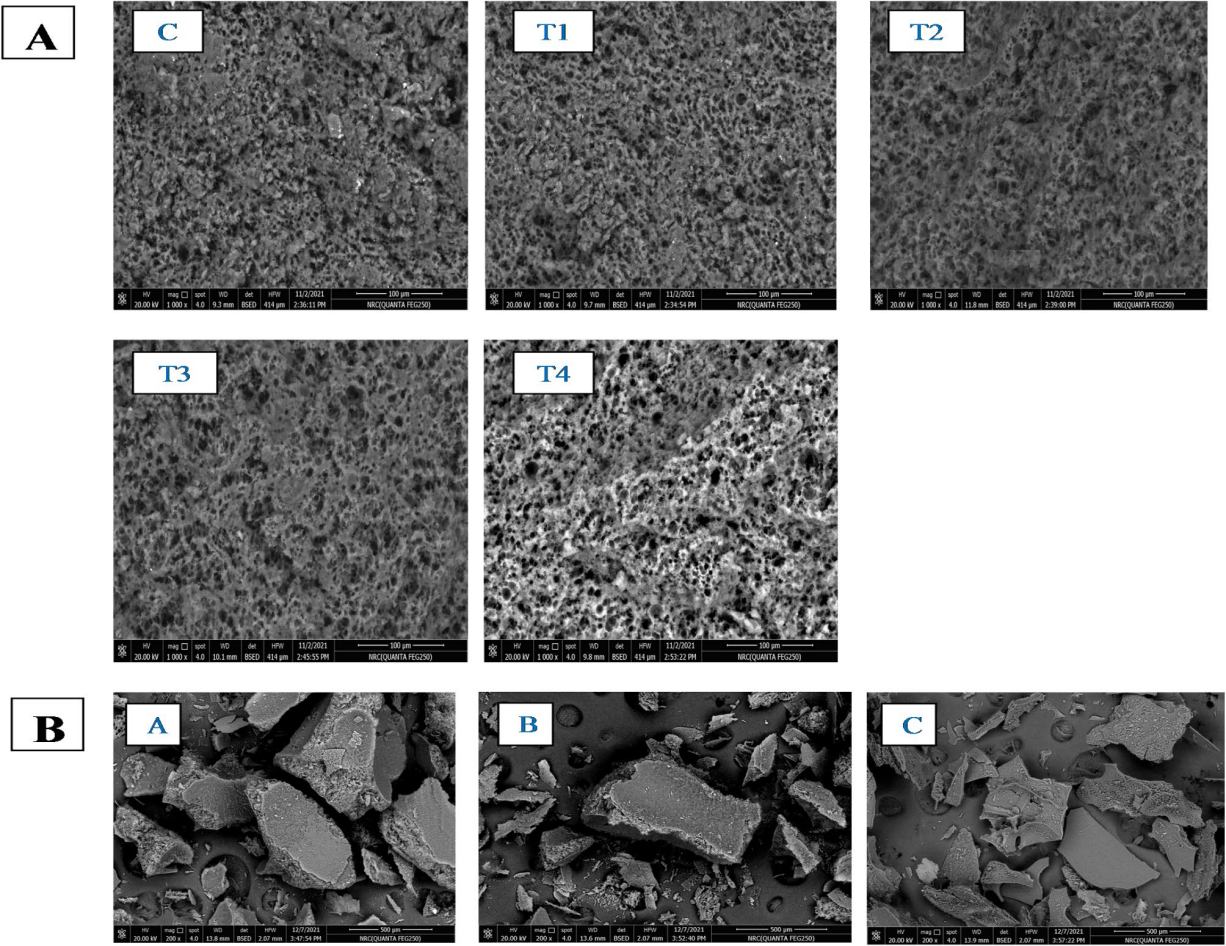
SEM micrographs. **A** UF-soft cheese fortified with free or encapsulated garlic extract using WPI/AG coacervateafter 7 days from storage at 5 ± 2 °C. **B** SEM micrographs of WPI/GA coacervate; **A** loaded with 80 mg GE; **B** loaded with 120 mg; **C** loaded with 160 mg

Figure 1B depicts the SEM micrographs of WPI/GA coacervate with different concentrations of GE. Microparticles of the EGE had an irregular-concave shape with no surface craters, fissures, or cracks. Microparticles with clearly defined surface features were created due to the intense interaction between WPI/GA. The degree of surface depression on the surface of microparticles may be due to the rapid drying and freezing that occurs during freeze-drying. Similar results from SEM images for polysaccharide and protein-based coatings from the freeze-drying of polyphenolic extract were reported by Papoutsis et al.^43^, who discovered that the interaction between the polyphenol and coating components caused different particle morphologies. Also, all the microparticles appeared agglomerated together due to their extreme humidity. The microparticles’ morphology remained unaffected by the strength of electrostatic forces, as evidenced by their strong agglomeration potential and lack of substantial alterations between samples. Similar results were obtained with the WPI/GA coacervate for omega-3-rich tuna oil, which showed that the particle surface lacked pores or cracks, protecting the encapsulated oil^14^.

### UF-soft cheese properties

#### Changes on soft cheese chemical composition

The UF retention of soft cheese composition, as verified by FGE and EGE, is displayed in Table 2. Compared to the control cheese, the composition of UF-soft cheese fortified with FGE was not different (*P* > 0.05). However, total solids and total proteins gradually increased as EGE increased, the increase being significant only at the addition of 3.35% EGE. This increase is related to an increase in WPI/GA coacervate levels. The total solids and total proteins increased from 32.06 ± 0.59 and 11.31 ± 0.22% in control cheese to 33.14 ± 1.65 and 34.33 ± 1.03%, and 11.94 ± 0.68 and 12.43 ± 0.79%, respectively, in cheese fortified with 1.67 and 3.35% EGE.

**Table 2:** Composition of UF-soft cheese fortified with free or encapsulated garlic extract using WPI/GA coacervate

Cheese treatments	Chemical composition			
	Total solids (%)	Total protein (%)	Fat (%)	Ash (%)
Control	32.06^b^ ± 0.59	11.31^b^ ± 0.22	13.0^a^ ± 0.00	4.03^a^ ± 0.34
T1	31.96^b^ ± 0.69	11.33^b^ ± 0.48	13.0^a^ ± 0.12	3.97^a^ ± 0.22
T2	32.21^b^ ± 0.41	11.32^b^ ± 0.46	13.2^a^ ± 0.50	3.99^a^ ± 0.19
T3	33.14^ab^ ± 1.65	11.94^ab^ ± 0.68	12.9^a^ ± 0.25	3.94^a^ ± 0.26
T4	34.33^a^ ± 1.03	12.43^a^ ± 0.79	12.9^a^ ± 0.25	3.95^a^ ± 0.33

Means (*n* = 3 ± SD) with the same letters in the same column are not significantly different (*P* < 0.05).

*T1* soft cheese fortified with 0.2% free garlic extract, *T2* soft cheese fortified with 0.4% free garlic extract, *T3* soft cheese fortified with 0.2% garlic extract in encapsulated form, *T4* soft cheese fortified with 0.4% garlic extract in encapsulated form.

The FGE fortified UF retentet soft cheese composition did not differ significantly from that of the control cheese (*P* > 0.05). But as EGE got higher, total solids and total proteins grew continuously; the rise became apparent only when 3.35% EGE was incorporated. The WPI/GA coacervate levels have gone up in correlation with the increase. Total solids and total proteins rose to 33.14 ± 1.65 and 34.33 ± 1.03%, and 11.94 ± 0.68 and 12.43 ± 0.79%, respectively, in cheese added with 1.67 and 3.35% EGE, from 32.06 ± 0.59 and 11.31 ± 0.22% in the control cheese. The chemical analysis of the soft white agreed with Saad et al.^44^. This may be due to the augmented proportion of the added plants extract as well as the percentage of the microcapsulat materials used to emulsify the extract and confirm its distribution in the final product.

#### Biochemical changes

Table 3 indicates that soft cheese’s pH decreased little upon the addition of FGE, but the pH significantly declined (*P* < 0.05) when FGE in the form of EGE had been added. Such an effect was seen by Abd El-Aziz et al.^21^ in fresh cheese incorporating ginger extract by ethanol. The acidic nature of phenolic compounds in FGE and their interaction with milk proteins, as well as the acidic characteristics of WPI/GA coacervate, may be responsible for the pH reduction^45,46^. The pH values of the cheese samples were decreased throughout storage, reaching a significant level only on day 30 (*P* < 0.05). Since glycolysis produces lactic acid, this can be used as an explanation for the pH reduction. However, the rate of reduction was slower in T1 and T2 compared to T3, T4, and control cheese, indicating that FGE showed a high antimicrobial effect during storage. Garlic or thyme extracts had the best ability to suppress molds and yeasts as well as to delay or prevent the production of mycotoxins. Such as allicin, that have been biologically active compounds in garlic, displays antibacterial, antifungal, antiparasitic, and antoxidant proberties^47,48^.

**Table 3:** Biochemical changes of UF-soft cheese fortified with free or encapsulated Garlic extract using WPI/GA coacervate during storage at 5 ± 2 °C for 30 days

Items	Storage period (day)	Soft cheese treatments				
		Control	T1	T2	T3	T4
pH	1	6.45^Aa^ ± 0.07	6.37^Aa^ ± 0.11	6.34^Aa^ ± 0.08	6.11^Ba^ ± 0.13	5.98^Ba^ ± 0.02
	15	6.16^Aa^ ± 0.14	6.19^Aa^ ± 0.02	6.15^Aab^ ± 0.01	5.88^Ba^ ± 0.17	5.74^Bab^ ± 0.14
	30	5.72^ABb^ ± 0.11	5.84^Ab^ ± 0.16	5.96^Ab^ ± 0.18	5.49^Bb^ ± 0.07	5.53^Bb^ ± 0.05
SN/TN ratio (%)	1	8.68^Bb^ ± 0.28	8.68^Bb^ ± 0.42	8.68^Bb^ ± 0.56	12.2^Ab^ ± 0.42	13.08^Ac^ ± 0.40
	15	10.47^Cb^ ± 0.66	11.05^Cab^ ± 0.3	11.4^Cab^ ± 0.41	14.65^Bb^ ± 0.23	18.26^Ab^ ± 1.41
	30	13.98^Ca^ ± 1.39	13.96^Ca^ ± 1.35	13.57^Ca^ ± 0.88	19.68^Aa^ ± 2.25	22.59^Aa^ ± 0.79
DPPH- radical-scavenging activity (%)	1	6.62^Cb^ ±1.53	11.85^Bb^ ± 0.77	16.26^Ab^ ± 1.34	15.78^Ab^ ± 153	16.50^Ab^ ± 1.05
	15	11.87^Ba^ ± 1.73	16.99^ABa^ ± 2.91	21.40^Aa^ ± 1.41	21.59^Aa^ ± 0.87	23.18^Aa^ ± 2.87
	30	13.64^Ba^ ± 2.01	20.81^Aa^ ± 0.68	22.12^Aa^ ± 0.57	22.73^Aa^ ± 2.31	25.14^Aa^ ± 1.43

Means (*n* = 3 ± SD) with the same capital letters in the same raw or the same small letters in the same column are not significantly different at *P* ≤ 0.05.

*T1* soft cheese fortified with 0.2% free garlic extract, *T2* soft cheese fortified with 0.4% free garlic extract, *T3* soft cheese fortified with 0.2% garlic extract in encapsulated form, *T4* soft cheese fortified with 0.4% garlic extract in encapsulated form.

The addition of FGE had no effect on the soft cheese’s WSN/TN ratio on first day storage, which is an indication of cheese proteolysis. Even so, due to the high whey protein concentration in the WPI/AG coacervate, the WSN/TN ratio of the cheese enriched with EGE was significantly higher than that of the cheese enriched with free GE (*P* < 0.05). During storage, the degree of proteolysis was continually increasing in all cheese samples; for T1, T2, T3, and control cheese, the increases were significant at day 30, but for T4, the changes were significant at day 15 and 30. Storage of Uf soft cheese had been more soluble nitrogen because of the process of proteolysis by both proteases as rennet enzymes, all types of starter bacteria added when manufactured^28,49,50^.

Many bioactive compounds found in GE, such as polyphenolic, flavonoids, alliin, allicin, diallyl sulphides, and others, are associated with the strong DPPH• radical scavenging^51^. The DPPH• radical scavenging assay showed that soft cheese fortified with FGE and EGE had higher antioxidant activity than control cheese (*P* < 0.05). The antioxidant proberties of UF soft cheese samples also gradually increased as storage time increased; the increase was significant on day 15 (*P* < 0.05). The soft cheese’s antioxidant activity develops with storage as a consequence of cheese proteolysis (r^2^ = 0.73)^52^. The release of bioactive peptides with low molecular weight during the ripening period of soft cheese resulted in a difference in antioxidant capacity between the soluble and nonsoluble fractions, as confirmed by Chen and Guangqing^53^ and Ramos et al.^54^. However, EGE exhibited more DPPH-radical scavenging activity than FGE at the same concentration due to the encapsulation technique showing highly-protected polyphenols against processing conditions during cheese making. Farrag et al.^55^ found that olive pomace polyphenols capsules in UF soft white cheese give high stability of free radicle scavenging. In particular, GE administered at 0.2% in either free form (T1) or encapsulated form (T3) resulted in a significant difference (*P* < 0.05).

#### Texture profile

Table 4 shows the texture profile of UF-soft cheese fortified with free or EGE during storage at 5 ± 2 °C for 30 days. Fortification of soft cheese with FGE and EGE was increased hardness and gumminess compared to control cheese; however, only EGE-fortified cheese showed a significant difference (*P* < 0.05). Similar observations were made by Soliman et al.^56^ in UF-cheese supplemented with encapsulated phenolic plant extracts. The increase can be attributed to several factors, including the reduction in pH induced by FGE, the usage of GA in the WPI/GA coacervation, and the increase in total solids, especially total proteins. Also, the pH of the cheese directly impacts the solubility of the caseins, which affects the curd’s texture; high-pH cheeses are softer than more acidic cheeses^57^. According to Solowiej^58^, whey proteins may have filled up gaps in the casein network or produced a mixed gel network. While α-lactalbumin and casein could form bonds, casein and β-lactoglobulin were the most disulfide-producing proteins. A smooth, uniform, and firm texture is produced by GA’s assistance in binding water and oil molecules^59^. The cohesiveness and gumminess of soft cheese were unaffected by the addition of FGE and EGE (*P* > 0.05). The hardness and springiness of the cheese also progressively increased during the course of storage, becoming significant only on day 30 (*P* < 0.05). The contraction of the milk gel and subsequent reorganization of the network caused by the attraction interactions between individual casein particles may be the cause of the rise in cheese hardness after storage^60^. The fight for water intensifies when more recent groups are generated new ionic of molecular peptides as synthesized during protein hydrolysis^21^. Therefore, less water may solvate the protein chains, resulting in a cheese that is less elastic and firmer. On the other hand, cheese cohesiveness progressively reduced during the course of storage; on day 30, the drop was only statistically significant in the T2, T3, and T4 groups (*P* < 0.05).

**Table 4:** Texture profile of UF-soft cheese fortified with free or encapsulated Garlic extract using WPI/GA coacervate

Items	Storage period (day)	Soft cheese treatments				
		Control	T1	T2	T3	T4
Hardness (N)	1	6.20^Bb^ ± 0.98	7.20^ABb^ ± 0.64	7.22^ABb^ ± 1.06	8.10^Ab^ ± 1.13	8.90^Ab^ ± 0.56
	15	8.20^Bb^ ± 0.85	9.10^ABb^ ± 1.13	9.30^ABb^ ± 0.99	10.30^Ab^ ± 0.38	10.75^Ab^ ± 0.35
	30	10.30^Ca^ ± 0.91	12.1^BCa^ ± 0.84	12.64^Ba^ ± 1.03	13.29^Ba^ ± 0.63	16.00^Aa^ ± 0.67
Cohesiveness	1	0.73^Aa^ ± 0.03	0.67^Aa^ ± 0.01	0.71^Aa^ ± 0.01	0.70^Aa^ ± 0.05	0.70^Aa^ ± 0.02
	15	0.70^Aa^ ± 0.02	0.65^Aa^ ± 0.09	0.66^Aa^ ± 0.03	0.73^Aa^ ± 0.02	0.69^4b^±0.01
	30	0.65^Aa^ ± 0.07	0.65^Aa^ ± 0.06	0.53^Ab^ ± 0.11	0.56^Ab^ ± 0.04	0.54^Ab^ ± 0.02
Springiness (mm)	1	0.76^Aa^ ± 0.04	0.79^Aa^ ± 0.02	0.76^Aa^ ± 0.03	0.76^Aa^ ± 0.04	0.73^Aa^ ± 0.05
	15	0.69^Aa^ ± 0.01	0.68^Aa^ ± 0.01	0.71^Aa^ ± 0.03	0.69^Aa^ ± 0.01	0.73^Aa^ ± 0.03
	30	0.72^Aa^ ± 0.05	0.70^Aa^ ± 0.01	0.63^Aa^ ± 0.02	0.68^Aa^ ± 0.01	0.70^Aa^ ± 0.07
Gumminess (N)	1	4.41^Bb^ ± 0.99	4.77^ABb^ ± 0.76	5.10^ABa^ ± 0.75	5.63^ABb^ ± 0.85	6.29^Ab^ ± 0.92
	15	5.21^Bab^ ± 0.45	5.43^Bab^ ± 0.23	6.43^ABa^ ± 0.51	7.49^Aab^ ± 0.97	7.38^Aab^ ± 0.74
	30	6.30^Ba^ ± 0.22	6.86^ABa^ ± 0.28	6.79^ABa^ ± 0.73	7.56^ABa^ ± 0.76	8.24^Aa^ ± 0.64

Means (*n* = 3 ± SD) with the same capital letters in the same raw or the same small letters in the same column are not significantly different at *P* ≤ 0.05.

*T1* soft cheese fortified with 0.2% free garlic extract, *T2* soft cheese fortified with 0.4% free garlic extract, *T3* soft cheese fortified with 0.2% garlic extract in encapsulated form, *T4* soft cheese fortified with 0.4% garlic extract in encapsulated form.

#### Microstructure of UF-soft cheese

As shown in Fig. 1A, several forms of UF-soft cheese, including control cheese (C), cheese fortified with 0.2 and 0.4% FGE (T1 and T2), or 0.2 and 0.4% FGE in encapsulated form (T3 and T4). A preliminary SEM study of the control Uf soft cheese exhibited an open, heterogeneous protein structure. The FGE-fortified cheese (T1 and T2) was more comparable to the control cheese; however, the outer surface of the FGE-soft cheese looked denser and had fewer gaps. These images could confirm the higher hardness and gumminess values of FGE-soft cheese than control cheese (Table 4). UF-treated cheeses may also have higher hardness due to a higher total solid content of the retentate and higher calcium retention in the milk^61^. EGE-fortified cheese (T3 and T4) showed rough sheets-like structures. The protein matrix produced an asymmetrical network because it was made up of protein granules of different sizes joined by fiber- or sheet-like appendages of different lengths. The structure is in line with other cheese varieties’ photos that have been published by other researchers^62^.

#### Color parameters

One of the key factors affecting food’s consumer attractiveness and, as a result, sales of the product, is its hue. The degree of cheese whiteness was not substantially impacted by the addition of FGE, as indicated in Table 5, but the degree of cheese greenness was reduced; the reduction was proportionate to the added amount (*P* > 0.05). Abd El-Aziz et al.^63^ detected this development in raw ground beef boosted with FGE and white cheese samples pickled in a brine solution with aqueous or ethanolic ginger extract^64^. Due to the FGE’s sunset-yellow hue, the yellowness degree was more obvious when it was added at a rate of 0.4% (T2). Garlic’s colour is made up of several pyrrole-based purple/blue and yellow species^65^. The reddish-purple pigment in garlic is a dipyrrole molecule, according to Imai et al.^66^. As the cross-linking process produces more tri- or tetrapyrrole molecules, a colour change from blue to green is to be anticipated. The presence of EGE, on the other hand, significantly reduced the whiteness and greenness of the cheese while increasing its yellowness (*P* < 0.05). The lessening of whiteness was in accordance with research by El-Sayed et al.^19^ which demonstrated that adding fig leaf extract in the form of capsules reduced the whiteness of processed cheese.

**Table 5:** Color parameters of UF-soft cheese fortified with free or encapsulated Garlic extract using WPI/GA coacervate

Items	Soft cheese treatments				
	Control	T1	T2	T3	T4
L*	89.56^A^ ± 1.24	90.69^A^ ± 1.13	89.69^A^ ± 1.06	86.06^B^ ± 1.36	83.89^B^ ± 0.95
a*	− 1.97^A^ ± 0.18	− 1.42^B^ ± 0.11	− 1.35^B^ ± 0.07	− 0.98^C^ ± 0.05	− 0.84^C^ ± 0.09
b*	12.03^B^ ± 0.79	12.38^AB^ ± 0.84	14.34^A^ ± 0.49	14.27^AB^ ± 0.86	16.06^AC^ ± 0.99

Means (*n* = 3 ± SD) with the same capital letters in the same raw are not significantly different at *P* ≤ 0.05.

*T1*, soft cheese fortified with 0.2% free garlic extract, *T2* soft cheese fortified with 0.4% free garlic extract, *T3* soft cheese fortified with 0.2% garlic extract in encapsulated form, *T4* soft cheese fortified with 0.4% garlic extract in encapsulated form, *L* darkness from black (0) to white (100), *a* color red (+) to green (−), *b* color yellow (+) to blue (−).

#### Sensory acceptability

The sensory characteristics scores of UF retentet of soft cheese enhanced with FGE or EGE for look, texture, taste, and overall quality are shown in Table 6. Fortification of UF-soft cheese with both FGE and EGE enhanced the texture characteristics on day 1; the improvement was significant when compared to control cheese on day 4 (*P* < 0.05). Compared to the control cheese, all cheese tretments had a smooth texture and a solid body. Additionally, the FGE and EGE-enriched cheese were superior to the control in terms of look, taste, and overall quality especially that fortified with 0.2% FGE in capsule form (T3). On day 30, all sensory attribute ratings dropped; the only sensory attribute where the drop was significant cheese appearance (*P* > 0.05).

**Table 6:** Sensory evaluation scores of UF-soft cheese fortified with free or encapsulated Garlic extract using WPI/AG coacervate

Items	Storage period (day)	Soft cheese treatments				
		Control	T1	T2	T3	T4
Appearance	1	8.11^a^ ± 0.78	8.33^a^ ± 0.50	8.25^a^ ± 0.83	8.25^a^ ± 0.66	8.12^ab^ ± 0.78
	30	7.25^b^ ± 1.21	7.25^b^ ± 0.83	7.22^b^ ± 0.93	7.13^bc^ ± 1.05	7.13^b^ ± 0.82
Texture	1	7.78^b^ ± 1.00	8.22^ab^ ± 0.66	8.37^ab^ ± 0.69	8.47^ab^ ± 0.70	8.62^a^ ± 0.48
	30	7.55^b^ ± 1.13	7.62^b^ ± 0.86	7.62^b^ ± 0.86	7.63^b^ ± 0.86	7.75^b^ ± 0.66
Flavor	1	8.7^ab^ ± 1.00	8.29^ab^ ± 0.78	7.87^ab^ ± 0.71	8.50^a^ ± 0.50	8.12^ab^ ± 1.05
	30	7.11^b^ ± 1.53	7.24^b^ ± 0.83	7.12^b^ ± 1.05	7.74^ab^ ± 0.83	7.50_ab_ ± 0.71
Overall quality	1	8.00^b^ ± 1.00	8.00^ab^ ± 1.00	8.50^a^ ± 0.87	8.62^a^ ± 0.69	8.37^ab^ ± 0.69
	30	7.44^b^ ± 1.13	7.50^b^ ± 0.87	7.74^ab^ ± 0.97	8.00^ab^ ± 0.86	7.50^b^ ± 0.71

Means (*n* = 3 ± SD) with the same letters are not significantly different at *P* ≤ 0.05.

*T1* soft cheese fortified with 0.2% free garlic extract, *T2* soft cheese fortified with 0.4% free garlic extract, *T3* soft cheese fortified with 0.2% garlic extract in encapsulated form, *T4* soft cheese fortified with 0.4% garlic extract in encapsulated form.

## Conclusion

Encapsulation of hydrophobic ethanolic garlic extract using a complex coacervation technique involving WPI and GA was successfully implemented for garlic extract to improve its stability, protect the antioxidant activity, and mask the garlic odor and taste. The applicability of EGE in UF-soft cheese was higher than that of FGE. The yellowness degree was more pronounced when FGE was compared with EGE, which can be attributed to the sunset yellow color of FGE. On the other hand, the addition of EGE caused a significant decrease in the whiteness and greenness of the cheese. In addition, the cheese fortified with FGE and EGE was preferable to the control in terms of appearance, flavor, and overall quality especially that fortified with 0.2% EGE. The encapsulation of polyphenols extracted from plants by WPI/GA complex-coacervates paved the way for the manufacturing of functional foods and food fortification. The successfully created coacervate powders that are also loaded with GE may be utilized as appropriate supplements to enhance any kind of solid or liquid food, including cheese, fermented dairy products and beverages.

## Data Availability

The data that support the findings of this study are available from the corresponding author upon reasonable request.
